# IS*982* and kin: new insights into an old IS family

**DOI:** 10.1186/s13100-020-00221-z

**Published:** 2020-07-04

**Authors:** Nancy Fayad, Mireille Kallassy Awad, Jacques Mahillon

**Affiliations:** 1 Laboratory of Food and Environmental Microbiology, Earth and Life Institute, Université Catholique de Louvain, Croix du Sud, 2 - L7.05.12, B-1348 Louvain-la-Neuve, Belgium; 2 Laboratory of Biodiversity and functional genomics, UR-EGP, Faculty of Science, Université Saint-Joseph de Beyrouth, Beirut, Lebanon

**Keywords:** Antibiotic resistance, Archaea, DDE motif, Insertion sequences, Transposase

## Abstract

Insertion sequences (IS) are ubiquitous transposable elements with a very simple organization: two inverted repeats flanking a transposase coding gene. IS*982* is one of 26 insertion sequence families known so far. With 70 registered members in the ISFinder database, this family remains somewhat unexplored, despite the association of many of its members with important features such as antibiotic resistance. IS*982* has a fairly simple organization with a mean length of ca. 1 Kb, two inverted repeats with conserved 5′ AC 3′ ends flanking a transposase coding gene and direct repeats of variable lengths. Its transposase has a RNAse-H like chemistry with an atypical DDE motif.

In this study, we first highlight the current knowledge on the IS*982* family by dissecting its registered members and their characteristics. Secondly, we bring new insights into this old, yet uncharted IS family, by exploring its registered elements, as well as the genomic and proteomic databases of bacterial and archaeal strains. This probing showed that the presence and distribution of this family goes far beyond the clear-cut registry of ISFinder database.

## Background

Insertion sequences (IS) are ubiquitous, autonomous prokaryotic transposable elements (TEs), displaying variable genomic copy numbers [1–3]. They have a simple organization, typically consisting of a transposase (TnpA) coding gene, flanked by two terminal inverted repeats (IR) [4]. IS have been classified into families, not only by comparing their protein sequences, but also according to their TnpA chemistry as well as their structural features, including length of terminal IR and direct repeats (DR) generated upon transposition [5]. Details on IS families can be found on ISFinder, an online database including more than 4000 elements, grouped into 26 families (https://www-is.biotoul.fr/; last Database Update: 2019-11-13; last accessed May 2020 [6];). While most elements pertain to defined families, some are still “orphans” and are designated as Not Classified Yet or “ISNCY”.

Transposase chemistry dictates the breaking and re-joining of the DNA fragment during transposition. There are four distinct types of enzymes classified by their catalytic domains. Nevertheless, one important characteristic is shared by the four groups: the hydrolysis of high-energy cofactors is not required for any of the mobility steps [7]. During transposition, a nucleophilic attack allows strand break and the formation of an active protein - DNA complex, also known as a “synaptic complex” or “transpososome”. The next step is either the duplication of the element, thus mobilizing the TE via a replicative mechanism (i.e. copy-paste [8];), or a second strand break to excise it, therefore employing a conservative one (i.e. cut-paste [9];). The first enzyme type corresponds to TnpA with an RNase H-like catalytic domain. The active site of these enzymes includes a three-residue catalytic constellation: DDE (most frequently) or DDD. Often, the catalytic triad is surrounded by conserved amino acids (aa) or amino acids sharing chemical properties, the most common being K/R residues located six/seven aa downstream of the E (DDE(N)_6/7_K/R). The second type contains the HUH (H for histidine and U for bulky hydrophobic residue) TnpA, active only on single strand DNA (ssDNA), and operating via a peel and paste mechanism [10]. The third and fourth types are Serine (S) and Tyrosine (Y) TnpA, respectively. These transposases both share many catalytic features with other site-specific recombinases such as invertases and resolvases [7, 11]. Interestingly, most conjugative transposons are also mobilized by these S- or Y-TnpA [12, 13].

Transposase genes are highly regulated either through intrinsic regulation at the transcriptional or translational level, or by the host itself, as suggested in the case of certain bacterial stresses [14]. In some cases, the TnpA is encoded by more than one Coding DNA Sequence (CDS) in the event of a “programmed ribosomal frameshifting”, which may occur in various forms (− 1, − 2, + 1 or + 2) depending on the motifs present in the nucleotide sequence of the gene, such as slippery codons or hairpin loops in the DNA [15, 16]. These events are apparently used by both prokaryotic and eukaryotic organisms to regulate TnpA synthesis [14, 17] whose coding genes have been found to be the most prevalent genes in nature [18]. Their density on bacterial chromosomes is generally below 3%, with some exceptions, such as *Bordetella pertussis*, in which a particularly an abundance of IS*481* elements is found on the chromosome [19]. As for plasmids, IS density can reach up to an extreme of 40% as in the case of the *Shigella flexneri* plasmid pW100 [20].

Among the known IS families, that of IS*982* has remained largely ignored. Although many of its members are associated with important features such as antibiotic resistance, little is known about this family, its peculiarities and mode of transposition. Therefore, the aim of this mini-review was to bring new insights into IS*982* family, from its early days to the most recent discoveries. For this purpose, an extensive literary review of IS*982* known elements is accompanied here by a bioinformatic approach.

## IS*982*: discovery and known elements

Twenty-five years ago, Yu and collaborators (1995) discovered the first IS*982* element in the lactose plasmid pSK11L of a *Lactococcus lactis* (*L. lactis)* strain, between the origin of replication and the *opp* (oligopeptide permease) gene cluster [21]. The second IS*982* element, IS*982B*, was characterized shortly after, as an IS-like element identified in plasmid pCIT264 of *L. lactis* subsp. *lactis* biovar *diacetylactis* [22]. The subsequent discovery and characterization of related elements in *Lactococcus* strains, such as IS*982C* [23], led to the grouping of these IS into one family, designated as the IS*982* family. This family was long thought to only consist of IS found in Lactococci (e.g. IS*Lgar2* and IS*Lgar3* [24, 25]). Currently, the IS*982* family contains 70 distinct elements in the ISFinder database, from 35 bacterial and archaeal genera belonging to 12 different taxonomic groups (Table 1). Host genera were distributed among 23 Gram-negative and 10 Gram-positive bacteria, as well as 2 archaea. The most dominant bacterial groups are *Firmicutes* and *Gamma-Proteobacteria*. IS*Ot4* has a disrupted TnpA and was excluded from further analysis, bringing the number of analyzed elements to 69.

**Table 1:** List of IS*982* elements from the ISFinder database; ^a^ original host in which the element was found. ^b^ Bacterial/archaeal group to which belongs the original host of the IS*982* element according to LifeMap [26] and the NCBI taxonomy. ^c^ length of the IS*982* element including transposase CDS, left and right inverted repeats. ^d^ length of the IS*982* TnpA protein and coordinates of its CDS. ^e^ length of identical nt in the left and right IR over the total length. ^f^ Direct repeats in the element’s original host/species. * = Disrupted transposase coding gene

Name	Origin^a^	Gram (+/−) or Archaea	Bacterial and Archeal host Group ^b^	IS Length (bp)^c^	TnpA Length (aa)^d^	IR (bp)^e^	DR (bp)^f^
IS*1187*	*Bacteroides fragilis*	–	*FCB Group*	1028	326 (34–1014)	21/22	7
IS*1592*	*Pasteurella trehalosi*	–	*Gamma-proteobacteria*	1027	294 (100–984)	18/18	2
IS1599	*Moraxella* sp.	–	*Gamma-proteobacteria*	1026	293 (102–983)	18/18	0
IS*19*	*Lactococcus lactis*	+	*Firmicutes*	1038	301 (111–1016)	21/21	9
IS*195*	*Porphyromonas gingivalis*	–	*FCB Group*	1070	300 (176–1078)	12/12	8
IS*233A*	*Bacillus thuringiensis*	+	*Firmicutes*	1028	302 (101–1009)	25/25	8
IS*982*	*Lactococcus lactis*	+	*Firmicutes*	999	296 (91–981)	19/20	8
IS*982B*	*Lactococcus lactis*	+	*Firmicutes*	999	296 (91–981)	19/20	8
IS*982C*	*Lactococcus lactis*	+	*Firmicutes*	999	296 (91–981)	19/20	0
IS*Aba4*	*Acinetobacter baumannii*	–	*Gamma-proteobacteria*	975	292 (90–968)	18/22	0
IS*Aba47*	*Acinetobacter baumannii*	–	*Gamma-proteobacteria*	972	279 (83–922)	17/20	7
IS*Aba6*	*Acinetobacter baumannii*	–	*Gamma-proteobacteria*	1003	301 (84–988)	12/12	6
IS*Aba825*	*Acinetobacter baumannii*	–	*Gamma-proteobacteria*	975	291 (85–960)	17/17	7
IS*Aba9*	*Acinetobacter baumannii*	–	*Gamma-proteobacteria*	974	293 (84–965)	17/17	8
IS*Acsp2*	*Acinetobacter* sp.	–	*Gamma-proteobacteria*	972	289 (91–960)	11/17	9
IS*Alw19*	*Acinetobacter lwoffii*	–	*Gamma-proteobacteria*	980	287 (95–958)	13/13	8
IS*Alw20*	*Acinetobacter lwoffii*	–	*Gamma-proteobacteria*	979	287 (95–958)	12/12	6
IS*Bs1*	*Bacillus stearothermophilus*	+	*Firmicutes*	996	290 (102–975)	18/21	0
IS*Caa5*	*Candidatus Amoebophilus*	–	*FCB Group*	920	274 (86–910)	21/25	6
IS*Cca1*	*Candidatus Cardinium*	–	*FCB Group*	1000	291 (105–980)	21/22	4
IS*Cca4*	*Candidatus Cardinium*	–	*FCB Group*	999	291 (104–979)	18/19	6
IS*Cef2*	*Corynebacterium efficiens*	+	*Actinobacteria*	1017	301 (84–989)	24/26	0
IS*Cef3*	*Corynebacterium efficiens*	+	*Actinobacteria*	989	301 (68–973)	11/11	0
IS*Clce1*	*Clostridium cellulovorans*	+	*Firmicutes*	1157	313 (115–1056)	11/13	0
IS*Cth1*	*Clostridium thermocellum*	+	*Firmicutes*	1151	315 (109–1056)	22/29	7
IS*Dds4*	*Deinococcus deserti*	+	*Deinococcus*	845	257 (65–838)	18/23	3
IS*Dds5*	*Deinococcus deserti*	+	*Deinococcus*	845	257 (65–838)	19/24	6
IS*Dge8*	*Deinococcus geothermalis*	+	*Deinococcus*	907	271 (55–870)	19/19	5
IS*Efm1*	*Enterococcus faecium*	+	*Firmicutes*	1041	302 (111–1020)	22/22	7
IS*Fba2*	*Flavobacteria bacterium*	–	*FCB Group*	998	277 (150–982)	18/18	0
IS*Ftu4*	*Francisella tularensis*	–	*Gamma-proteobacteria*	963	284 (92–945)	11/11	7
IS*Gsp1*	*Geobacillus* sp.	+	*Firmicutes*	1004	292 (105–983)	14/14	9
IS*Gth1*	*Geobacillus thermodenitrificans*	+	*Firmicutes*	1001	292 (102–980)	14/14	9
IS*Lbp3*	*Leptospira borgpetersenii*	–	*Spirochaetes*	1115	305 (185–1102)	25/32	0
IS*Lgar2*	*Lactococcus garvieae*	+	*Firmicutes*	998	296 (90–980)	20/20	8
IS*Lgar3*	*Lactococcus garvieae*	+	*Firmicutes*	994	297 (99–992)	16/17	6
IS*Lh1*	*Lactobacillus helveticus*	+	*Firmicutes*	962	271 (137–952)	32/35	7
IS*Lhe1*	*Lactobacillus helveticus*	+	*Firmicutes*	965	240 (78–800)	27/28	8
IS*Lhe5*	*Lactobacillus helveticus*	+	*Firmicutes*	965	285 (98–955)	27/28	0
IS*Lhe7*	*Lactobacillus helveticus*	+	*Firmicutes*	965	285 (98–955)	27/28	8
IS*Lla2*	*Lactococcus lactis*	+	*Firmicutes*	998	296 (90–980)	21/27	0
IS*Lll1*	*Lactococcus lactis*	+	*Firmicutes*	999	296 (91–981)	18/20	4
IS*Lpl4*	*Lactobacillus plantarum*	+	*Firmicutes*	985	292 (97–975)	29/35	8
IS*Neu1*	*Nitrosomonas europaea*	–	*Beta-proteobacteria*	996	292 (97–972)	18/18	8
IS*Neu2*	*Nitrosomonas europaea*	–	*Beta-proteobacteria*	1000	208 (350–973)	21/21	3
IS*Nsp1*	*Nostoc* sp.	–	*Cyanobacteria/Melainabacteria*	1028	297 (121–1011)	16/17	6
ISOt4*	*Orientia tsutsugamushi*	–	*Alpha-proteobacteria*	988	251 (226–980)	15/18	5
IS*Pasp1*	*Parachlamydia* sp.	–	*PVC Group*	1018	292 (124–999)	17/17	5
IS*Pasp2*	*Parachlamydia* sp.	–	*PVC Group*	1023	292 (128–1003)	16/17	4
IS*Pasp3*	*Parachlamydia* sp.*.*	–	*PVC Group*	952	275 (105–929)	22/22	10
IS*Pfu3*	*Pyrococcus furiosus*	Archaea	*Thermococci*	933	289 (59–930)	15/16	2
IS*Plu11*	*Photorhabdus luminescens*	–	*Gamma-proteobacteria*	988	294 (91–975)	15/17	2
IS*Plu6*	*Photorhabdus luminescens*	–	*Gamma-proteobacteria*	990	294 (93–977)	15/17	0
IS*Prsp2*	*Parabacteroides* sp.	–	*FCB Group*	1021	324 (33–1007)	21/22	7
IS*Psa1*	*Piscirickettsia salmonis*	–	*Gamma-proteobacteria*	1282	239 (282–1001)	09/18	7
IS*Psma1*	*Psychrobacter maritimus*	–	*Gamma-proteobacteria*	983	294 (90–974)	20/21	3
IS*Ra1*	*Riemerella anatipestifer*	–	*FCB Group*	983	292 (4688–5566)	14/16	0
IS*Rmsp1*	*Ruminococcus* sp.	+	*Firmicutes*	1112	312 (92–1030)	21/28	9
IS*Sa4*	*Streptococcus agalactiae*	+	*Firmicutes*	962	287 (90–953)	24/24	9
IS*Scr1*	*Streptococcus criceti*	+	*Firmicutes*	962	287 (89–952)	22/25	6
IS*Sde7*	*Shewanella denitrificans*	–	*Gamma-proteobacteria*	989	295 (89–976)	12/17	8
IS*Smu5*	*Streptococcus mutans*	+	*Firmicutes*	951	297 (49–942)	17/18	0
IS*Sod20*	*Shewanella oneidensis*	–	*Gamma-proteobacteria*	995	292 (104–982)	15/20	9
IS*Spy2*	*Streptococcus pyogenes*	+	*Firmicutes*	962	287 (89–952)	21/23	8
IS*Ssu12*	*Streptococcus suis*	+	*Firmicutes*	962	302 (44–952)	27/28	2
IS*Sude1*	*Sulfurimonas denitrificans*	–	*Epsilon-proteobacteria*	916	279 (75–914)	15/16	0
IS*Tli1*	*Thermococcus litoralis*	Archaea	*Thermococci*	905	280 (58–902)	15/16	2
IS*Vsa6*	*Aliivibrio salmonicida*	–	*Gamma-proteobacteria*	1001	293 (95–976)	17/17	6
IS*Wpi16*	*Wolbachia pipientis*	–	*Alpha-proteobacteria*	981	290 (95–967)	18/18	0
IS*Xne5*	*Xenorhabdus nematophila*	–	*Gamma-proteobacteria*	980	294 (93–977)	20/26	7

**Fig. 1:**
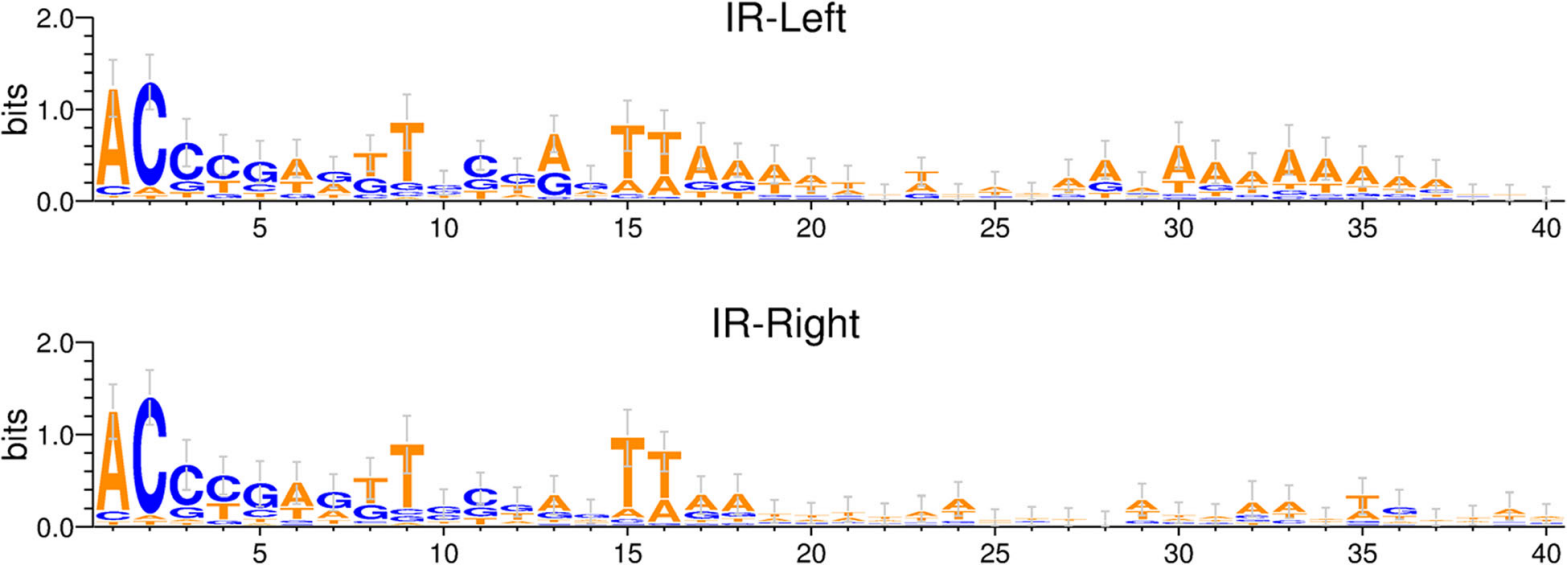
IS*982* inverted repeats left and right sequence logo, generated by WebLogo 3 [28]. The x-axis represents position of the corresponding nt. The y-axis represents bits, which indicate the maximum entropy for the given sequence type (log_2_ 4 = 2 bits for DNA/RNA). The height of symbols within the stack reflects the relative frequency of the corresponding nt at that position

The size of IS*982* family members ranges between 845 and 1282 bp, with a mean length of 996 bp. By convention, the left and right IR are located upstream and downstream of TnpA transcriptional unit, respectively. Their IR are between 11 and 32 identical bp. DR were either already reported in ISFinder or retrieved by manual search via nucleotide BLAST (BLAST.N) of the element against the original host/species genome sequence. When present, DR range from 2 to 10 bp (Table 1). This variation in DR length is not unusual and was previously reported in other families such as IS*4* (4–13 bp, [27]). An extreme case of DR variability was also reported in the ISFinder database: IS*1182* family with DR ranging between 2 and 60 bp. A multiple sequence alignment of the left and right IR (Additional Fig. S1) showed that the most conserved positions are 5′-AC(N)_6_T(N)_5_TT-3′ ends, as shown in Fig. 1. Out of 69 analyzed elements, 57 begin with “AC”, and 5 with CC.

IS*982* family members can be quite divergent and originate from many different species. However, some elements are isoforms, presenting high sequence identity (> 95% DNA; > 98% protein) such as IS*19* and IS*Efm1* from *Enterococcus faecium*, or IS*982*, IS*982B* and IS*982C* from *Lactococcus lactis*. While the location, copy number and potential association with host genes have been reported for several IS*982* family members, some elements, such as IS*Dds4* and IS*Dds5,* were simply annotated following whole genome sequencing without further information. An example of a well-described element is IS*Ra1,* found on a *Riemerella anatipestifer* plasmid that contains the *vapD* gene, thought to encode a virulence factor, in two to twenty copies in some strains of this species [29]. Another example is IS*Lh1,* present in multiple copies on the chromosome and on one plasmid of *Lactobacillus helveticus* strains [30]. For other characterized IS*982* family elements, such as IS*1187*, IS*Lpl4*, IS*195* and IS*Efm1*, copy number ranged between one and ten copies, distributed between chromosomes and plasmids [31–33].

## Transposase groups within IS*982* family

As indicated above, members of the IS*982* family are widely distributed within the prokaryotic world. The current host range spans from Gram-negative bacteria to archaea and includes intracellular bacteria. This wide host range, combined with pairwise TnpA sequence identities spanning from ca. 25 to 98% highlights the extent of divergence within this family. Additional analysis of pairwise distance estimation with Poisson correction between IS*982* family elements, using Mega-X [34] with a ClustalW algorithm, corroborates this divergence (Additional Table S1). This estimation highlights the possibility of aa substitution in a certain position of the protein. A smaller value entails a closer relationship and less divergent sequences. To further investigate the diversity of IS*982* family and the relationship between its members and their hosts, a genetic tree was constructed based on the comparison of their transposases. Following a MAFFT alignment of their protein sequences [35], a dendrogram of relationship between IS*982* TnpA was constructed by a neighbor-joining method (NJ) using a JTT model [36], with a bootstrap value of 500. The tree was rooted with the clade containing the two archaeal IS*982* family elements, that were the most divergent in this family following pairwise alignment.

**Fig. 2:**
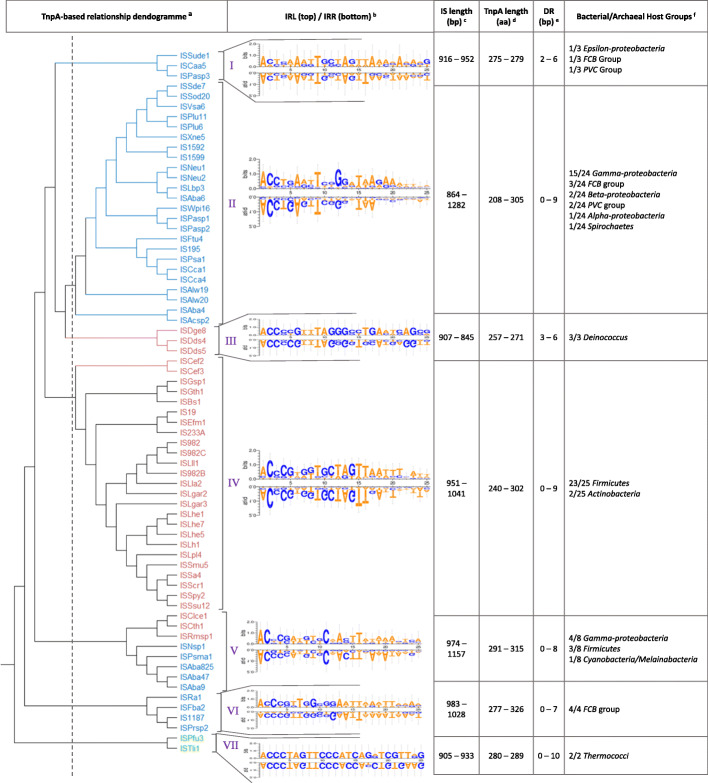
**a** Dendrogram representing the relationship among the 69 IS*982* transposases. Protein sequences were first aligned with MAFFT, and the relationship tree was established via neighbor joining, with a bootstrap value of 100, followed by a rooting [35]. Blue, pink and green colors refer to the IS original hosts as Gram-negative bacteria, Gram-positive bacteria and archaea, respectively; **b** WebLogo [28] 3 comparison of the left (top) and right (bottom) IR of each cluster; **c** complete IS element nt length range (bp); **d** transposase protein length range (aa); **e** Direct repeats length range (bp) and **f** count of bacterial and archaeal groups within each cluster

As shown in Fig. 2, most, but not all, IS*982* family elements originally found in Gram-negative or Gram-positive bacteria tend to cluster together. In some cases, clustering of elements originating from the same species (e.g. IS*Lhe1*, IS*Lhe7*, IS*Lhe5* and IS*Lh1* from *Lactobacillus helveticus*), the same genus (e.g. IS*Dds4,* IS*Dds5,* IS*Dge8* from *Deinococcus* spp.), or the same host group (e.g. 15 elements originating from *Gamma-proteobacteria*), is evident and might reflect the early presence of these elements in the evolution of their hosts. In other cases, there is a great distance between elements originating from the same species or genus. For instance, IS*Aba4*, IS*Aba47*, IS*Aba6*, IS*Aba825* and IS*Aba9* from *Acinetobacter baumannii* (*A. baumannii*) strains are distant from each other.

Figure 2 also shows several deep branches within the transposase tree of IS*982* family elements. Setting the threshold of TnpA protein sequence identity at 35% (dotted line; Fig. 2), seven clusters (I to VII) could be identified. This division is coherent not only with the aforementioned closeness of elements from the same host species, genus or group, but also with the conservation of IR sequences within each cluster.

Many families in the ISFinder database are divided into groups or sub-families, based on TnpA protein sequence identity, IR length and ends as well as DR length [4]. IS982 family is no different, and this cluster division may allow the ISFinder team to define clear sub-families within IS982.

## IS*982* family transposase structure and chemistry

IS*982* family elements present the typical simple IS organization of two terminal IRs flanking a transposase coding gene. The corresponding TnpA contains on average ca. 290 residues. Although most transposases originate from a single CDS, this is not always the case. For instance, IS*Tli1*, found in the archaeon *Thermococcus litoralis* [37], displays two CDSs which, through a − 1 frameshifting event, will result in a single 280 aa enzyme, whose activity was not proven experimentally. Another example is that of the functional IS elements IS*Lpl4*. Interestingly, in this case, the two CDSs were shown to be fused by a + 1 frameshifting event, the first described case of a functional + 1 frameshifting among bacterial IS at the time [38]. IS*Lpl4* pattern (copy number and single nucleotide polymorphisms) changed over generations of the original *Lactobacillus plantarum* strain, CECT 4645, indicating that this IS was active at one or multiple times in the strain’s evolution. Nonetheless, the study by De Las Rivas et al. (2005) proved the functionality of the + 1 frameshift, by using the *lacZ* as a reporter gene. The fusion of the frameshifting site with the reporter gene gave a low 1.5% β-galactosidase activity [35].

**Fig. 3:**
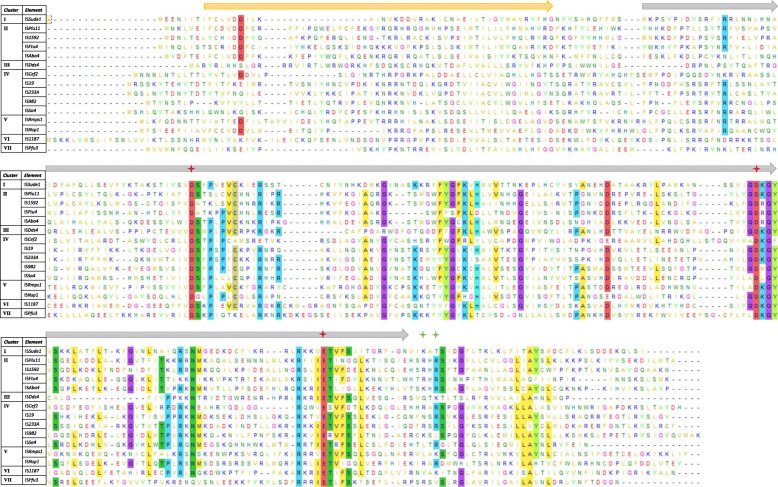
Multiple Sequence alignment of 15 randomly chosen IS*982* elements from the seven clusters. Alignment was done using Mega-X via a ClustalW algorithm [34]. Only residues conserved at a minimum of 50% are highlighted. Predicted helix-turn-helix and DDE domains are indicated by orange and grey arrows. The catalytic triad DDE and the potential missing K/R residue are indicated by red and green marks, respectively

Although their mechanism is yet to be unraveled, previous studies pointed out that IS*982* family transposases carry a DDE motif [35]. Yet, unlike other described DDE TnpA so far, they do not present a conserved K/R residue six/seven aa downstream of the catalytic glutamate, earning it the label of an atypical DDE motif [4]. However, a semi-conserved K/R residue was detected further downstream, after ca. 17 aa, just outside of the predicted DDE domain (Fig. 3). As for other DDE domains, the three catalytic acidic residues (two aspartate and a glutamate) are thought to initiate a nucleophilic attack on a phosphodiester bond of the donor DNA [7]. What follows is either replicative (copy/paste) or conservative (cut/paste) transposition to a target DNA site.

A TnpA multiple sequence alignment of 15 randomly selected IS*982* elements from the seven clusters revealed several conserved aa, alongside the catalytic DDE triad, as shown in Fig. 3 (For an alignment of all elements, see additional Fig. S2). A noteworthy observation is that many conserved aa are located in regions flanked by the aspartate and glutamate of the DDE motif. IS*982* family transposases possess a predicted Helix-turn-Helix motif at the N-terminal of the TnpA, acting as DNA-interacting domain with the IR sequences, upstream of the predicted DDE domain, which spans more than 60% of the protein.

No crystal structure for any IS*982* family TnpA is available yet. However, secondary structure predictions using the RaptorX-Property tool [39] revealed an abundance of helical structures (ca. 40%) (data not shown). Also, although the TnpA chemistry of a catalytic triad is conserved throughout all IS*982* elements, some differences may arise at the level of DNA-TnpA interaction during DNA transfer (cut/paste, copy/paste or co-integrate) among the different elements form the seven clusters due to the observed divergence TnpA protein sequence.

## Exploring the genome meta-database

ISFinder is well-established database of “clean” IS elements, beyond which is a large genomic world of bacterial and archaeal strains that we set out to further explore for the presence and distribution of IS*982* family members. Therefore, archaeal and bacterial protein databases were mined for possible new IS*982*-like elements by a protein BLAST (BLAST.P) search, using the default parameters, with a cut-off of 50% query coverage (QC) and 30% identity (ID) and a maximum target sequence number of 1000. The results were a high number of hits, from which a sample is described below. In the newly found elements, the DDE motif was highly conserved.

This approach remains limited in finding novel IS elements, since its reach is lesser than that of PSI-BLAST, which compiles BLAST. P hits as a search matrix to find more distant results. However, PSI-BLAST results, although comparable to those of BLAST. P, would require more verification, the subject of a future study. The following section is exploratory, scratching the surface of bacterial groups holding IS*982*-related elements.

### IS982-like elements in archaea

Knowledge about the archaeal super-kingdom, an important part of Earth’s microbiome, is ever changing since new meta-genomic, meta-transcriptomic and meta-proteomic datasets, metabolic predictions and phylogenetic assessments are being derived [40, 41]. This super-kingdom is, so far, divided into four main super-phyla: Euryarchaeota, TACK, DPANN and the recently described Asgards [42] (http://lifemap-ncbi.univ-lyon1.fr/; [26]; last update: December 2019).

In 2007, a review by Filée et al. highlighted what was known about the diversity of IS in archaea at the time, undoubtedly confirming that this group of organisms is an intriguing source of TEs. Several IS families were found within the available genomes, with IS*Pfu3* from *Pyrococcus furiosus* [43, 44] being the only archaeal IS*982* family member. More recently, IS*Tli1* was found in *Thermococcus litoralis* [37]. *Pyrococcus* and *Thermococcus* both belong to the *Thermococcales* order. As shown in Fig. 2, a large distance separates the branch holding IS*Pfu3* and IS*Tli1* from the rest of the bacterial IS*982* family elements. This suggests that these elements were likely not transferred from archaea to bacteria, or vice-versa.

Protein transposases sequences of IS*Pfu3* and IS*Tli1* were used to probe the archaeal database by BLAST. P searches. Six potential new IS*982* elements were found in *Methanotorris formicicus* (5) and *Methanocaldococcus bathoardescencs* (1), two Euryarchaeota species, belonging to the *Methanococcales* order, indicating that the IS*982* family members are not restricted to the *Thermococcales*. Hits with the bacterial protein database did not pass the established thresholds of 50% QC and 30% ID, reinforcing the distance of IS*982* family elements from archaeal or bacterial origins.

### IS982-like elements in bacteria

For the bacterial IS*982* family elements, five representative elements were chosen from distant branches of the IS*982* transposase relationship dendrogram (Fig. 2). BLAST. P searches were conducted against the bacterial and archaeal non-redundant protein databases. Only hits with bacterial strains were found.

#### Elements originally from gram-negative strains

Three IS*982* elements out of the five selected ones are IS*Pasp3* from *Parachlamydia*, IS*Ftu4* from *Francisella* and IS*1187* from *Bacteroides* [31]. The original Gram-negative hosts of these three IS are classified in different bacterial groups according to the NCBI taxonomy and Lifemap [26]: *Parachlamydia* is a part of the PVC group (*Planctomycetes*, *Verrucomicrobia*, and *Chlamydiae*), *Francisella* belongs to the *Gamma-Proteobacteria* group and *Bacteroides* is part of the *FCB* group (*Fibrobacteres*, *Chlorobi* and *Bacteroidetes*). BLAST. P analysis showed that the resulting hits were quite diverse for each IS, as shown in Table 2. For the three elements, a total of 265 new genera containing IS*982*-related elements were found. Among these genera, 37.7% belong to the *FCB* group, 16.2% to *Gamma-Proteobacteria* and 14.3% to *Cyanobacteria*.

**Table 2:** Distribution of IS*982*-related elements based on a BLAST. P search

Element	Original host genus (bacterial/archaeal group)	Total number of genera*	Number of new genera (%)**	Most prevalent group in the new genera (%)
**IS*Pfu3***	*Pyrococcus* (Archaea; *Thermococcales*)	4	2 (50%)	*Thermococcales* (62.5%)
**IS*Tli1***	*Thermococcus* (Archaea; *Thermococcales*)	4	2 (50%)	*Thermococcales* (86.3%)
**IS*Pasp3***	*Parachlamydia* (Gram-negative; *PVC* group ^a^)	80	75 (93.75%)	*FCB* group (42.6%)
**IS*Ftu4***	*Francisella* (Gram-negative; *Gamma-proteobacteria*)	140	125 (89.3%)	*Gamma-proteobacteria* (31.2%); *cyanobacteria* (24.8%); *FCB* group (23.2%)
**IS*1187***	*Bacteroides* (Gram-negative; *FCB* group ^b^)	132	120 (90.9%)	*FCB* group (57.5%)
**IS*Cef2***	*Corynebacterium* (High GC Gram-positive; *Actinobacteria*)	81	79 (97.5%)	High GC Gram+ (86%)
**IS*Cth1***	*Clostridium thermocellum* (Gram-positive; *Firmicutes*)	133	20 (85%)	*Firmicutes* (30.8%); *FCB* group (31.5%)

* Number of genera where the specific IS982-related element was found

** Genera not previously known to hold IS*982*-related elements

^a^ PVC group: *Planctomycetes*, *Verrucomicrobia*, and *Chlamydiae* group

^b^ FCB group: *Fibrobacteres*, *Chlorobi* and *Bacteroidetes* group

#### Elements originally from gram positive strains

As for IS originating from Gram-positive hosts, IS*Cef2* from *Corynebacterium efficiens*, a high GC Gram-positive bacteria in the Actinobacteria group [45] and IS*Cth1* from *Clostridium thermocellum*, belonging to the *Firmicutes* group, were considered for BLAST. P searches (Table 2). In total, 180 new genera were identified, 36.3, 19.5 and 17.9% of which are classified as high GC Gram-positive bacteria, *FCB* group bacteria and *Firmicutes*, respectively. A noteworthy observation was that ca. 42.16% of hits obtained with IS*Cef2* originated from *Streptomyces* strains.

## IS*982* elements, friends or foes?

IS*982* family elements, like all IS, can affect the donor as well as the target site, during transposition. In the following section, an overview of the possible effects of known IS*982* family members on genes present on the target site, is presented. The discussed elements and their effects are summarized in Table 3.

**Table 3:** Consequences of the insertion of IS*982* family elements into the promotor region (A) or the coding DNA sequence (B) of antibiotic resistance or virulence genes. The effects include the complete or partial activation/increase of expression (↑) or inactivation (↓) of the corresponding gene(s)

Insertion site	Element	Effect	Affected gene	Consequences
A. Within the promotor region	IS*Aba4*	↑	*bla*_*OXA*_ carbapenemase	Resistance to carbapenem
	IS*Aba9*	↑		
	IS*Aba47*	↑		
	IS*Aba825*	↑		
	IS*1187*	↑	*cfiA*	Resistance to carbapenem
	IS*Lhe1*	↓ Partial	Between *lacL* and *lacR*	Reduction of β-galactosidase activity
B. Within the CDS	IS*19*	↓	D-Alanine:D-Alanine ligase	Resistance to vancomycin and teicoplanin
	IS*Efm1*	↓	D-Alanine:D-Alanine ligase	Resistance to vancomycin and teicoplanin
	IS*Aba825*	↓	*carO*	Resistance to carbapenem
	IS*Sa4*	↓	*cylB*	Loss of virulence
	IS*Scr1*	↓	*paaB*	Loss of virulence
	IS*Bs1*	↓	*glgB*	Negative impact on the cell metabolism and physiology
	IS*195*	↓ Partial	Cysteine protease	Disruption of its Arg-X cleavage ➔ Decrease in virulence

### Antibiotic resistance

The emergence of antibiotic resistant bacteria is becoming a major threat to the environment as well as the human health. IS*982* family has its fair share of elements associated with antibiotic resistance genes. Such is the case of IS*19* and IS*Efm1* from *Enterococcus faecium*, inserted after transposition into the D-Alanine:D-Alanine ligase coding gene. Disruption of the corresponding ligase leads to the absence of the D-Alanine:D-Alanine precursors, and the presence of only those ending in D-Alanyl:D-Lactate. Consequently, these bacteria became resistant to vancomycin [33] and teicoplanin [46], two antibiotics that act specifically on the aforementioned D-Alanine:D-Alanine and inhibit cell wall synthesis. Another example of gene disruption associated with antibiotic resistance is that of IS*Aba825* from *A. baumannii,* whose insertion inactivates *carO,* a gene encoding a transmembrane protein thought to participate in the influx of carbapenem. This led to the development of *A. baumannii* strains resistant to carbapenem. An interesting observation was also made regarding the difference in the GC content between IS*Aba825* and its chromosomal insertion site, suggesting an exogenous origin of this element [47].

Another way for IS*Aba825* to induce carbapenem resistance is by forming a hybrid promoter and activating, directly and indirectly, the expression of the OXA-type carbapenemases coding genes (*bla*_*OXA*_), responsible for carbapenem and imipenem (β-lactam antibiotics) resistance [48–50]. Along the same lines, the *bla*_*OXA*_ gene expression was enhanced following the insertion of IS*Aba4*, IS*Aba47* and IS*Aba9,* other identified IS*982* family members, in *A. baumannii* [51–54].

IS*1187*, found in a carbapenem resistant *Bacteroides fragilis* strain, also induces antibiotic resistance by gene activation. *CfiA* is a Carbapenemase coding gene, conferring resistance to practically all β-lactams. The insertion of IS*1187* upstream of this normally promoter-less gene, provided − 7 and − 33 motifs, thus forming a mobile *Bacteroides* promoter allowing the production of Carbapenemase [31].

Certain elements are not directly responsible for antibiotic resistance but are possibly implicated in the plasmid-mediated phenotype. This is the case of IS*1592* located on pCCK13698, a 14.9 kb *Pasteurella trehalosi* plasmid which carries the *floR* gene, a florfenicol and chloramphenicol resistance gene. This plasmid is thought to be the result of several recombination events, in which IS*1592* could be involved [55].

IS*1599* and IS*Psma1* are other elements merely associated with, but not directly causing, antibiotic resistance. The former is present on a *Moraxella* sp. plasmid with tetracycline resistance [56] and the latter is on a plasmid carrying five antibiotic resistance genes, pKLH80, from *Psychrobacter maritimus* [57]. All listed examples reflect the high implication of this family in antibiotic resistance development.

A counter effect of insertion of an IS*982* family element may be antibiotic susceptibility. This was reported in *Enterococcus faecium* where the *liaF* gene, part of the LiaFSR operon, encoding stress response regulatory systems, was disrupted by an IS*982* family element. The disruption of *liaF* led to the reversion of daptomycin resistance to hyper susceptibility in the strain [58].

### Reduction of bacterial virulence

Gene disruption might also lead to changes in the cell metabolism, possibly affecting its growth and ecology. For instance, IS*Sa4* is responsible for the loss of the hemolytic activity of *Streptococcus agalactiae (S. agalactiae).* Among the 15 IS*Sa4* copies present in a specific strain, one was inserted in *cylB*, a gene encoding the membrane-spanning domain of the putative hemolysin transporter. IS*Sa4* could be detected only in strains isolated after 1996, which might indicate a recent acquisition of this novel insertion element by *S. agalactiae* [59]. Also in Streptococci, IS*Scr1* interrupts a *paaB* gene in the downstream region of the *antigen I/II* gene in *Streptococcus cricetus* [60, 61]. Antigen I/II is a key element in mediating the attachment of the bacterial cell to host components and in determining cell surface properties [62]. Another example is IS*Bs1* that disrupts *glgB* (glycogen branching enzyme) coding gene in a strain of *Bacillus stearothermophilus* [63]. This enzyme plays a crucial role in carbon and energy storage, therefore affecting the cell metabolism and physiology [64].

In some cases, however, certain IS*982* family members cause a reduction in activity in lieu of a total loss. An example is the IS*Lhe1* element from *L. helveticus.* Its location between the *lacL* and *lacR* genes in the lactose gene cluster may account for a reduced β-galactosidase activity in this strain [65]. Another example involves IS*195* found in *Porphyromonas gingivalis*. Its insertion within a cysteine protease coding gene led to a disruption of its Arg-X cleavage site specificity, thus a massive decrease in the virulence and infectious capacities of this strain [32].

## Conclusion

In this mini-review, the unexplored IS*982* family was studied by investigating its known elements, their origins as well as their structural and chemical properties. In addition, the extent of this family beyond ISFinder was demonstrated.

IS*982* harbors 70 members registered in the ISFinder database. They are ca. 1 kb in length, have IR starting with conserved 5′-AC(N)_6_T(N)_5_TT-3′ ends and carry a gene encoding an RNase-H like transposase with an atypical DDE motif. Exploring the genomic and proteomic databases via protein BLAST searches showed the immense number and variety of elements this family has yet to offer, in bacteria as well as in archaea, keeping in mind the impact IS*982* family members can have on antibiotic resistance or virulence, as highlighted in this study. Nevertheless, the precise mode of transposition of IS*982* family members remains unknown. Therefore, an in-depth analysis must take place to uncover the detailed transposition pathway of this old family that still has much hidden.

## Supplementary information

**Additional file 1: Figure S1.** The first (Left End) and complement of the last (Right End) 25 nt of IS*982* elements, ordered according to the relationship dendrogram shown in Fig. 2. Each nucleotide is colored differently.

**Additional file 2: Figure S2.** Multiple Sequence alignment of IS*982* family transposases, ordered according to the relationship dendrogram (Fig. 2). Alignment was done using Mega-X via a ClustalW algorithm [34]. Only residues conserved at a minimum of 50% are highlighted. Predicted helix-turn-helix and DDE domains are indicated by orange and grey arrows. The catalytic triad DDE and the potential missing K/R residue are indicated by red and green marks, respectively.

**Additional file 3: Table S1.** Pairwise distance estimation with Poisson correction between IS*982* family elements, ordered according to the relationship dendrogram shown in Fig. 2. Elements belonging to each cluster (indicated on the left) are highlighted. These numbers reflect the possibility of aa substitution in a certain position of the protein, in a pairwise manner. For example, distance estimation values vary between zero and ca. 2.128. The higher the number, protein sequence identity percentage is lower.

## Data Availability

The datasets used and/or analyzed during the current study are available from the corresponding author on reasonable request. Additional data are available at BMC Genomics online.

## Abbreviations

IS: Insertion Sequences
TEs: Transposable Elements
TnpA: Transposase
IR: Inverted Repeats
DR: Direct Repeats
DDE/D: Aspartate Aspartate Glutamate/Aspartate
HUH: H for histidine and U for bulky hydrophobic residue
S- / Y-TnpA: Serine−/Tyrosine- transposase
K/R: Lysine/Arginine
QC: Query Coverage
ID: Identity
*L. lactis*: *Lactococcus lactis*
aa: Amino Acids
*A. baumannii*: *Acinetobacter baumannii*
*S. agalactiae*: *Streptococcus agalactiae*
BLAST.P/.N: BLAST Protein/Nucleotide
