# Supplementary material for: IS982 and kin: new insights into an old IS family
Source: Mob DNA. 2020 Jul 4;11:24. doi: 10.1186/s13100-020-00221-z (PMC7335449; doi:10.1186/s13100-020-00221-z)
Supplement: Supplementary file 1 — Additional file 1: Figure S1. The first (Left End) and complement of the last (Right End) 25 nt of IS982 elements, ordered according to the relationship dendrogram shown in Fig. 2. Each nucleotide is colored differently. [file 13100_2020_221_MOESM1_ESM.pdf]

Left End

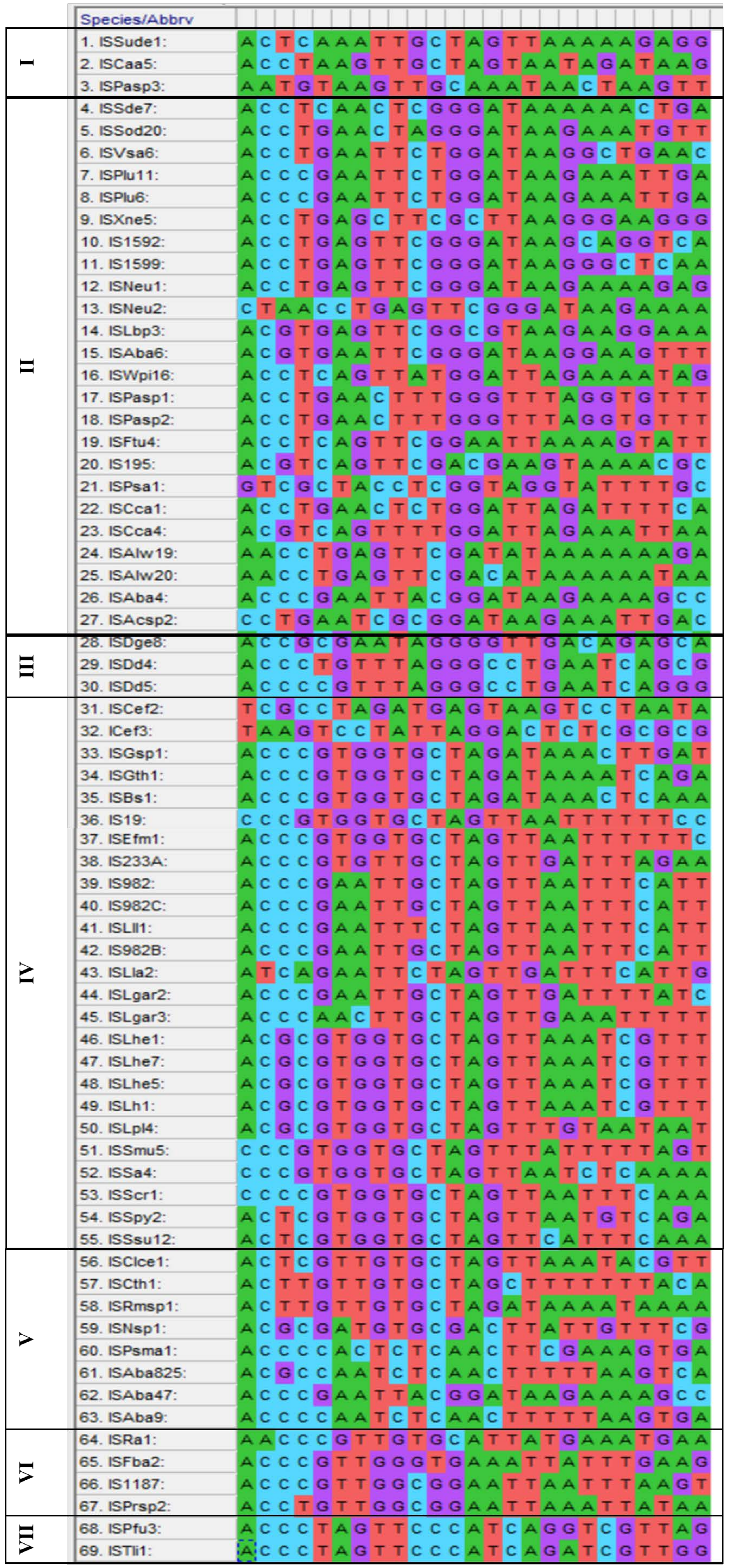

Right End

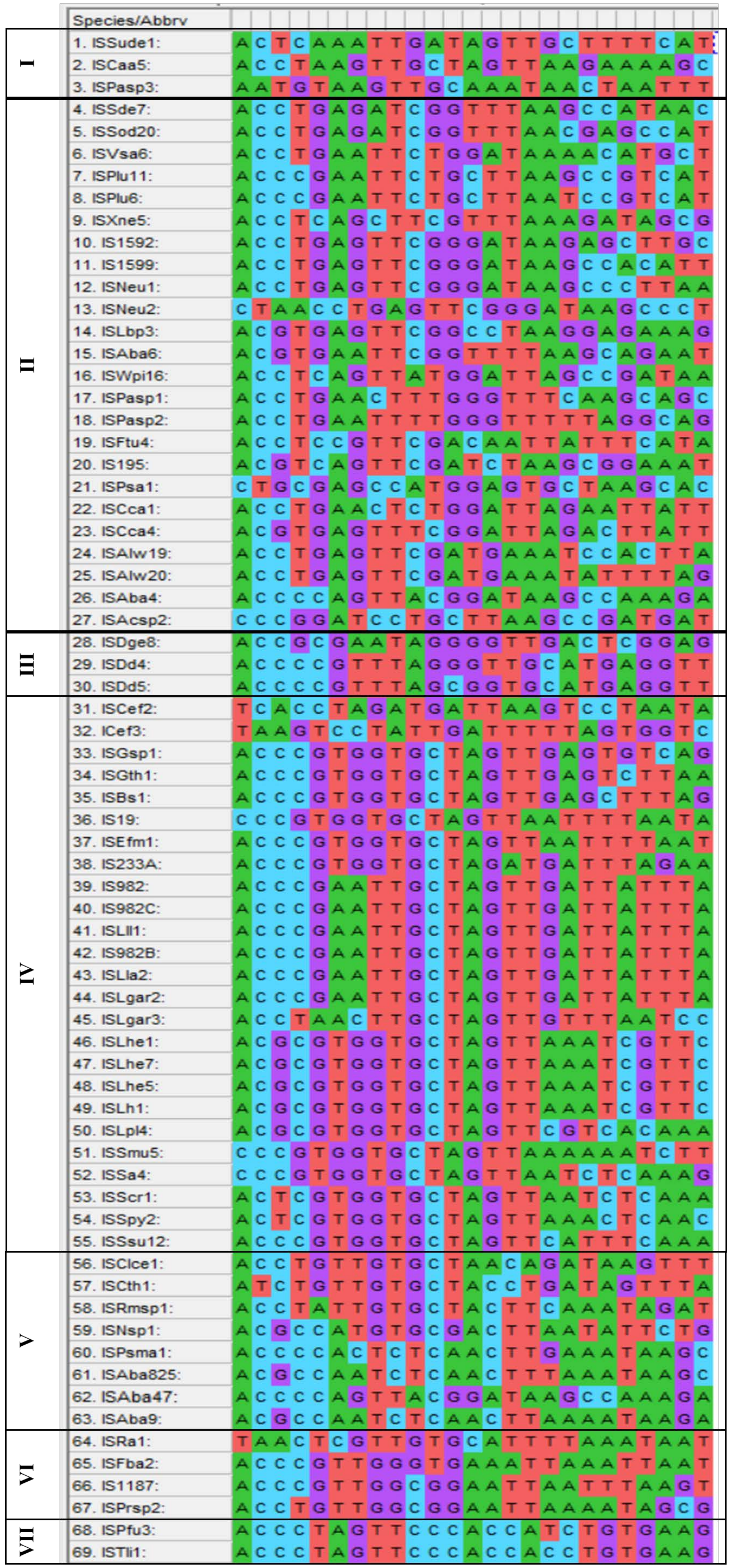

Figure S1
