# Supplementary figures and images for: IS982 and kin: new insights into an old IS family
Source: Mob DNA. 2020 Jul 4;11:24. doi: 10.1186/s13100-020-00221-z (PMC7335449; doi:10.1186/s13100-020-00221-z)

Figure S2

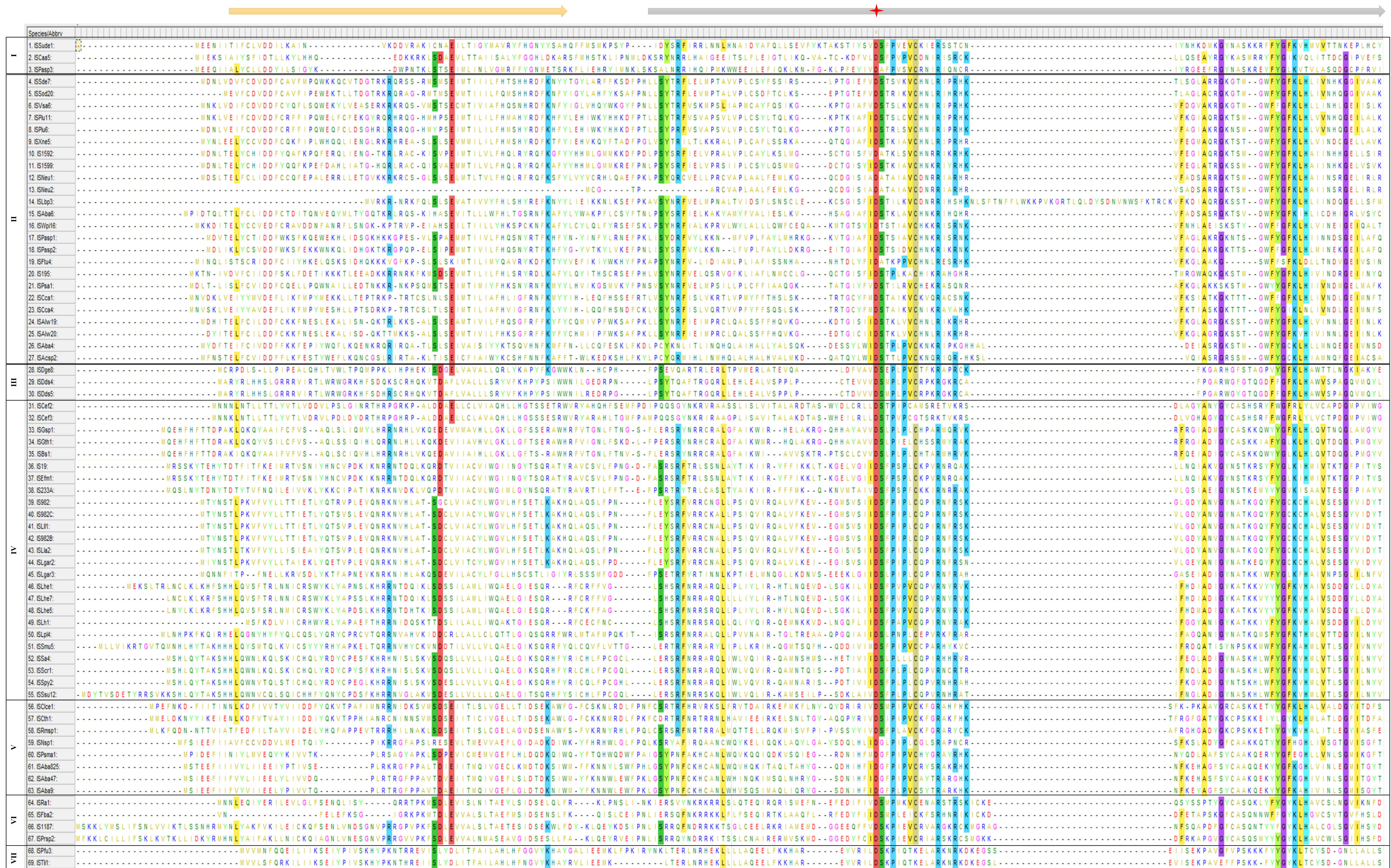

[illegible]

Supplement: Supplementary file 2 — Additional file 2: Figure S2. Multiple Sequence alignment of IS982 family transposases, ordered according to the relationship dendrogram (Fig. 2). Alignment was done using Mega-X via a ClustalW algorithm [34]. Only residues conserved at a minimum of 50% are highlighted. Predicted helix-turn-helix and DDE domains are indicated by orange and grey arrows. The catalytic triad DDE and the potential missing K/R residue are indicated by red and green marks, respectively. [file 13100_2020_221_MOESM2_ESM.pdf]
