# Supplementary material for: IS982 and kin: new insights into an old IS family
Source: Mob DNA. 2020 Jul 4;11:24. doi: 10.1186/s13100-020-00221-z (PMC7335449; doi:10.1186/s13100-020-00221-z)
Supplement: Supplementary file 3 — Additional file 3: Table S1. Pairwise distance estimation with Poisson correction between IS982 family elements, ordered according to the relationship dendrogram shown in Fig. 2. Elements belonging to each cluster (indicated on the left) are highlighted. These numbers reflect the possibility of aa substitution in a certain position of the protein, in a pairwise manner. For example, distance estimation values vary between zero and ca. 2.128. The higher the number, protein sequence identity percentage is lower. [file 13100_2020_221_MOESM3_ESM.pdf]

Cluster		ISSude1:	ISCaa5:	ISPasp3:	ISSde7:	ISSod20:	ISVsa6:	ISPlu11:	ISPlu6:	ISXne5:	IS1592:	IS1599:	ISNeu1:	ISNeu2:	ISLbp3:	ISAba6:	ISWpi16:	ISPasp1:	ISPasp2:
Cluster I	ISSude1:																		
	ISCaa5:	1.24																	
	ISPasp3:	1.19	1.05																
Cluster II	ISSde7:	1.48	1.54	1.70															
	ISSod20:	1.55	1.60	1.77	0.22														
	ISVsa6:	1.55	1.61	1.68	0.52	0.56													
	ISPlu11:	1.48	1.56	1.58	0.61	0.59	0.55												
	ISPlu6:	1.45	1.61	1.55	0.62	0.59	0.55	0.13											
	ISXne5:	1.60	1.54	1.60	0.63	0.65	0.69	0.52	0.55										
	IS1592:	1.46	1.56	1.50	0.79	0.80	0.78	0.65	0.63	0.63									
	IS1599:	1.46	1.66	1.59	0.79	0.83	0.77	0.76	0.73	0.70	0.33								
	ISNeu1:	1.64	1.67	1.68	0.87	0.84	0.76	0.88	0.83	0.73	0.62	0.67							
	ISNeu2:	1.58	1.51	1.62	0.92	0.86	0.76	0.92	0.86	0.73	0.64	0.72	0.09						
	ISLbp3:	1.63	1.42	1.67	0.85	0.85	0.79	0.76	0.76	0.72	0.68	0.76	0.72	0.74					
	ISAba6:	1.48	1.60	1.74	1.02	0.98	0.94	0.95	0.94	0.94	0.87	0.89	0.89	0.92	0.95				
	ISWpi16:	1.43	1.72	1.69	1.00	0.94	0.92	0.90	0.86	0.84	0.90	0.87	0.87	0.84	0.83	0.96			
	ISPasp1:	1.45	1.49	1.57	0.81	0.80	0.75	0.76	0.73	0.70	0.79	0.74	0.76	0.78	0.66	1.02	0.74		
	ISPasp2:	1.44	1.49	1.57	0.82	0.81	0.69	0.74	0.74	0.69	0.76	0.77	0.79	0.82	0.66	1.02	0.75	0.32	
	ISFtu4:	1.64	1.52	1.61	1.06	1.13	0.97	1.02	1.03	0.94	0.97	1.03	0.98	0.97	0.89	1.07	0.99	0.93	0.90
	IS195:	1.55	1.55	1.64	0.83	0.86	0.82	0.85	0.81	0.80	0.76	0.80	0.80	0.77	0.62	1.06	0.85	0.80	0.79
	ISPsa1:	1.42	1.65	1.54	0.83	0.87	0.81	0.83	0.78	0.86	0.82	0.82	0.96	1.05	0.66	0.96	0.92	0.81	0.79
	ISCca1:	1.51	1.59	1.77	0.80	0.77	0.76	0.79	0.80	0.80	0.86	0.91	0.87	0.83	0.71	1.05	0.84	0.78	0.73
	ISCca4:	1.46	1.56	1.88	0.82	0.82	0.77	0.77	0.81	0.78	0.86	0.88	0.86	0.84	0.78	1.06	0.91	0.82	0.80
	ISAlw19:	1.53	1.61	1.55	0.80	0.77	0.69	0.77	0.72	0.72	0.75	0.76	0.64	0.66	0.76	0.94	0.85	0.74	0.75
	ISAlw20:	1.49	1.57	1.56	0.81	0.77	0.73	0.82	0.78	0.75	0.81	0.80	0.65	0.64	0.77	0.95	0.88	0.76	0.77
	ISAba4:	1.44	1.50	1.62	1.07	1.11	1.02	0.99	0.98	0.97	1.06	1.06	1.11	1.11	1.06	1.12	1.09	1.06	1.06
	ISAcsp2:	1.49	1.41	1.58	1.10	1.09	1.11	1.03	1.02	1.00	0.98	1.07	1.03	1.07	1.17	1.08	1.12	1.13	1.07
Cluster III	ISDge8:	1.73	1.70	1.77	1.75	1.76	1.68	1.82	1.84	1.86	1.87	1.86	1.75	1.73	1.80	1.83	1.81	1.80	1.85
	ISDds4:	1.70	1.92	1.93	1.77	1.74	1.75	1.68	1.75	1.75	1.71	1.87	1.70	1.55	1.85	1.91	1.79	1.91	1.89
	ISDds5:	1.77	1.92	1.95	1.77	1.74	1.73	1.70	1.73	1.77	1.71	1.90	1.75	1.63	1.88	1.91	1.84	2.03	1.97
Cluster IV	ISCef2:	1.80	1.61	1.75	1.76	1.68	1.80	1.80	1.82	1.59	1.69	1.72	1.65	1.62	1.75	1.70	1.77	1.72	1.72
	ISCef3:	1.80	1.61	1.87	1.78	1.73	1.87	1.99	2.01	1.74	1.76	1.72	1.70	1.73	1.82	1.74	1.83	1.86	1.76
	ISGsp1:	1.42	1.66	1.68	1.52	1.45	1.53	1.38	1.39	1.45	1.41	1.44	1.52	1.37	1.47	1.59	1.58	1.43	1.46
	ISGth1:	1.32	1.60	1.60	1.50	1.50	1.61	1.38	1.41	1.47	1.41	1.47	1.55	1.43	1.45	1.61	1.59	1.49	1.54
	ISBs1:	1.36	1.59	1.71	1.49	1.45	1.58	1.37	1.40	1.51	1.46	1.49	1.64	1.42	1.41	1.58	1.57	1.45	1.52
	IS19:	1.42	1.66	1.66	1.58	1.69	1.65	1.54	1.54	1.73	1.65	1.61	1.79	1.62	1.47	1.62	1.62	1.54	1.68
	ISEfm1:	1.42	1.66	1.66	1.58	1.69	1.65	1.54	1.54	1.73	1.65	1.61	1.79	1.62	1.47	1.62	1.62	1.54	1.68
	IS233A:	1.49	1.74	1.79	1.57	1.57	1.59	1.52	1.49	1.55	1.55	1.53	1.66	1.45	1.50	1.73	1.47	1.54	1.61
	IS982:	1.23	1.41	1.62	1.62	1.58	1.61	1.50	1.54	1.53	1.52	1.61	1.57	1.42	1.48	1.47	1.54	1.54	1.55
	IS982C:	1.24	1.39	1.62	1.60	1.55	1.57	1.46	1.51	1.50	1.49	1.57	1.55	1.38	1.43	1.44	1.53	1.49	1.50
	ISLll1:	1.23	1.39	1.60	1.60	1.57	1.57	1.45	1.50	1.48	1.47	1.55	1.54	1.36	1.43	1.43	1.53	1.50	1.52
	IS982B:	1.23	1.39	1.62	1.60	1.55	1.56	1.45	1.50	1.50	1.47	1.55	1.54	1.36	1.41	1.43	1.51	1.50	1.52
	ISLla2:	1.22	1.39	1.60	1.62	1.58	1.61	1.48	1.50	1.53	1.50	1.55	1.54	1.36	1.46	1.46	1.56	1.49	1.52
	ISLgar2:	1.16	1.45	1.62	1.55	1.52	1.54	1.46	1.46	1.50	1.49	1.49	1.54	1.38	1.43	1.44	1.54	1.47	1.47
	ISLgar3:	1.29	1.53	1.69	1.58	1.68	1.72	1.60	1.65	1.67	1.66	1.64	1.70	1.53	1.63	1.69	1.58	1.57	1.55
	ISLhe1:	1.38	1.44	1.63	1.52	1.55	1.54	1.40	1.42	1.54	1.43	1.45	1.50	1.30	1.28	1.51	1.53	1.47	1.51
	ISLhe7:	1.37	1.51	1.64	1.48	1.50	1.52	1.39	1.42	1.50	1.40	1.43	1.48	1.34	1.34	1.49	1.49	1.49	1.47
	ISLhe5:	1.36	1.49	1.62	1.48	1.50	1.52	1.42	1.45	1.48	1.37	1.40	1.47	1.32	1.39	1.46	1.47	1.51	1.49
	ISLh1:	1.34	1.52	1.65	1.43	1.39	1.45	1.31	1.36	1.48	1.45	1.43	1.45	1.32	1.42	1.45	1.51	1.48	1.45
	ISLpl4:	1.39	1.76	1.88	1.47	1.39	1.44	1.42	1.41	1.42	1.41	1.41	1.41	1.29	1.62	1.42	1.62	1.51	1.46
	ISSmu5:	1.41	1.75	1.78	1.51	1.46	1.56	1.35	1.38	1.52	1.41	1.42	1.42	1.30	1.54	1.47	1.57	1.57	1.57
	ISSa4:	1.45	1.57	1.74	1.49	1.44	1.44	1.32	1.32	1.46	1.35	1.41	1.41	1.32	1.50	1.46	1.60	1.36	1.40
	ISScr1:	1.45	1.61	1.78	1.51	1.49	1.54	1.35	1.35	1.54	1.44	1.50	1.41	1.28	1.52	1.49	1.60	1.39	1.40
	ISSpy2:	1.46	1.65	1.78	1.41	1.38	1.36	1.25	1.28	1.47	1.35	1.39	1.38	1.28	1.41	1.43	1.55	1.36	1.36
	ISSsu12:	1.45	1.59	1.76	1.47	1.44	1.49	1.34	1.32	1.49	1.39	1.47	1.47	1.34	1.50	1.51	1.53	1.34	1.34
Cluster V	ISClce1:	1.44	1.56	1.62	1.43	1.42	1.35	1.39	1.46	1.48	1.39	1.39	1.47	1.37	1.35	1.45	1.57	1.46	1.46
	ISCth1:	1.45	1.66	1.64	1.45	1.47	1.42	1.37	1.42	1.50	1.49	1.57	1.66	1.54	1.46	1.63	1.55	1.51	1.42
	ISRmsp1:	1.30	1.54	1.55	1.54	1.57	1.54	1.60	1.64	1.59	1.52	1.52	1.62	1.53	1.50	1.43	1.65	1.58	1.57
	ISNsp1:	1.40	1.61	1.61	1.43	1.38	1.45	1.37	1.37	1.31	1.45	1.45	1.32	1.36	1.39	1.37	1.40	1.37	1.27
	ISPsma1:	1.56	1.78	1.83	1.42	1.39	1.44	1.51	1.48	1.47	1.48	1.45	1.42	1.33	1.35	1.54	1.46	1.40	1.26
	ISAba825:	1.56	1.76	1.81	1.47	1.43	1.43	1.43	1.40	1.44	1.47	1.53	1.40	1.32	1.34	1.51	1.36	1.42	1.31
	ISAba47:	1.60	1.74	1.78	1.48	1.49	1.49	1.40	1.37	1.44	1.44	1.54	1.41	1.29	1.35	1.52	1.35	1.36	1.29
	ISAba9:	1.63	1.72	1.85	1.54	1.55	1.51	1.45	1.42	1.60	1.46	1.59	1.52	1.44	1.37	1.57	1.42	1.48	1.36
Cluster VI	ISRa1:	1.36	1.49	1.49	1.57	1.55	1.54	1.61	1.64	1.62	1.49	1.59	1.68	1.47	1.64	1.62	1.71	1.59	1.47
	ISFba2:	1.49	1.60	1.73	1.77	1.60	1.61	1.50	1.53	1.67	1.54	1.63	1.64	1.57	1.55	1.51	1.61	1.59	1.46
	IS1187:	1.68	1.75	1.71	1.65	1.63	1.60	1.70	1.65	1.74	1.58	1.69	1.81	1.76	1.79	1.63	1.75	1.55	1.52
	ISPrsp2:	1.57	1.70	1.67	1.74	1.67	1.72	1.72	1.72	1.65	1.57	1.57	1.70	1.60	1.71	1.64	1.75	1.52	1.57
Cluster VII	ISPfu3:	1.55	1.73	1.69	1.71	1.73	1.80	1.61	1.59	1.71	1.69	1.77	1.73	1.66	1.70	1.77	1.94	1.57	1.59
	ISTli1:	1.60	1.73	1.78	1.71	1.74	1.80	1.63	1.61	1.69	1.71	1.75	1.69	1.64	1.75	1.73	1.95	1.57	1.63

	ISFtu4:	IS195:	ISPsa1:	ISCca1:	ISCca4:	ISAlw19:	ISAlw20:	ISAba4:	ISAcsp2:	ISDge8:	ISDds4:	ISDds5:
IS195:	0.98											
ISPsa1:	0.89	0.74										
ISCca1:	0.95	0.79	0.81									
ISCca4:	1.01	0.87	0.87	0.20								
ISAlw19:	0.90	0.78	0.90	0.84	0.89							
ISAlw20:	0.92	0.78	0.84	0.82	0.87	0.09						
ISAba4:	1.25	1.15	1.13	1.00	0.99	1.07	1.11					
ISAcsp2:	1.25	1.18	1.14	1.14	1.13	1.04	1.07	0.48				
ISDge8:	1.82	1.85	1.83	1.91	1.99	1.81	1.86	1.75	1.74			
ISDds4:	1.96	1.71	2.12	1.82	1.89	1.79	1.79	1.74	1.79	0.83		
ISDds5:	1.96	1.76	2.05	1.92	2.01	1.74	1.84	1.79	1.79	0.81	0.09	
ISCef2:	1.75	1.92	1.85	1.75	1.73	1.72	1.80	1.71	1.76	1.95	2.13	2.13
ISCef3:	1.77	1.92	1.88	1.83	1.81	1.76	1.80	1.81	1.72	1.82	2.03	2.03
ISGsp1:	1.71	1.51	1.54	1.59	1.57	1.39	1.37	1.55	1.46	1.61	1.61	1.59
ISGth1:	1.71	1.48	1.52	1.57	1.54	1.59	1.57	1.53	1.46	1.67	1.69	1.67
ISBs1:	1.66	1.54	1.51	1.53	1.51	1.56	1.51	1.47	1.49	1.67	1.64	1.62
IS19:	1.83	1.61	1.66	1.67	1.62	1.51	1.54	1.49	1.50	1.96	1.81	1.78
ISEfm1:	1.83	1.61	1.66	1.67	1.62	1.51	1.54	1.49	1.50	1.96	1.81	1.78
IS233A:	1.73	1.67	1.69	1.60	1.62	1.53	1.51	1.60	1.53	1.91	1.78	1.81
IS982:	1.77	1.52	1.61	1.61	1.61	1.57	1.48	1.40	1.47	1.69	1.69	1.67
IS982C:	1.73	1.49	1.59	1.54	1.56	1.54	1.45	1.40	1.47	1.67	1.67	1.65
ISLll1:	1.71	1.47	1.59	1.56	1.56	1.54	1.45	1.37	1.44	1.67	1.67	1.65
IS982B:	1.71	1.47	1.57	1.54	1.54	1.54	1.45	1.39	1.45	1.67	1.67	1.65
ISLla2:	1.69	1.49	1.63	1.53	1.54	1.52	1.45	1.42	1.48	1.69	1.65	1.65
ISLgar2:	1.69	1.49	1.61	1.53	1.48	1.49	1.40	1.34	1.42	1.71	1.67	1.65
ISLgar3:	1.75	1.62	1.70	1.60	1.61	1.55	1.53	1.43	1.52	1.59	1.52	1.58
ISLhe1:	1.64	1.67	1.55	1.50	1.48	1.44	1.40	1.62	1.53	1.84	1.76	1.70
ISLhe7:	1.63	1.60	1.50	1.49	1.45	1.40	1.40	1.53	1.41	1.77	1.69	1.63
ISLhe5:	1.59	1.60	1.54	1.50	1.47	1.39	1.40	1.62	1.44	1.67	1.61	1.57
ISLh1:	1.57	1.48	1.53	1.37	1.34	1.34	1.28	1.46	1.32	1.71	1.55	1.55
ISLpl4:	1.75	1.53	1.72	1.58	1.59	1.42	1.39	1.59	1.55	1.52	1.46	1.46
ISSmu5:	1.66	1.44	1.67	1.40	1.41	1.38	1.39	1.61	1.53	1.62	1.59	1.59
ISSa4:	1.62	1.36	1.58	1.60	1.53	1.32	1.26	1.63	1.53	1.71	1.61	1.63
ISScr1:	1.62	1.44	1.62	1.56	1.51	1.39	1.35	1.63	1.55	1.67	1.63	1.61
ISSpy2:	1.60	1.38	1.58	1.49	1.46	1.35	1.30	1.57	1.48	1.67	1.57	1.57
ISSsu12:	1.58	1.47	1.69	1.56	1.56	1.44	1.39	1.61	1.52	1.59	1.47	1.47
ISClce1:	1.59	1.33	1.29	1.53	1.57	1.53	1.52	1.51	1.42	1.57	1.54	1.56
ISCth1:	1.59	1.48	1.49	1.43	1.50	1.51	1.56	1.48	1.47	1.65	1.65	1.65
ISRmsp1:	1.68	1.47	1.54	1.53	1.56	1.60	1.57	1.51	1.55	1.50	1.61	1.55
ISNsp1:	1.52	1.39	1.40	1.33	1.34	1.33	1.34	1.43	1.51	1.73	1.87	1.90
ISPsma1:	1.55	1.46	1.46	1.43	1.46	1.49	1.40	1.66	1.75	1.59	1.72	1.77
ISAba825:	1.53	1.37	1.40	1.41	1.43	1.36	1.32	1.62	1.52	1.64	1.61	1.69
ISAba47:	1.48	1.31	1.34	1.43	1.37	1.44	1.32	1.64	1.52	1.73	1.69	1.72
ISAba9:	1.60	1.41	1.34	1.54	1.50	1.41	1.31	1.60	1.57	1.64	1.65	1.67
ISRa1:	1.77	1.56	1.63	1.58	1.58	1.52	1.45	1.57	1.55	1.60	1.50	1.54
ISFba2:	1.74	1.61	1.74	1.58	1.58	1.51	1.48	1.54	1.49	1.56	1.38	1.43
IS1187:	1.74	1.82	1.64	1.67	1.78	1.76	1.70	1.61	1.73	1.58	1.50	1.46
ISPrsp2:	1.80	1.60	1.66	1.68	1.72	1.70	1.62	1.53	1.69	1.66	1.53	1.55
ISPfu3:	1.87	1.83	1.82	1.79	1.84	1.65	1.69	1.90	1.80	1.68	1.69	1.71
ISTli1:	1.86	1.86	1.83	1.82	1.87	1.61	1.65	1.88	1.85	1.71	1.69	1.72

	ISCef2:	ISCef3:	ISGsp1:	ISGth1:	ISBs1:	IS19:	ISEfm1:	IS233A:	IS982:	IS982C:	ISLll1:	IS982B:	ISLla2:	ISLgar2:
ISCef3:	0.29													
ISGsp1:	1.53	1.60												
ISGth1:	1.49	1.61	0.25											
ISBs1:	1.44	1.57	0.22	0.29										
IS19:	1.71	1.77	1.10	1.06	1.12									
ISEfm1:	1.71	1.77	1.10	1.06	1.12	0.00								
IS233A:	1.73	1.81	1.05	0.99	1.05	0.56	0.56							
IS982:	1.64	1.70	1.12	1.10	1.11	0.99	0.99	1.00						
IS982C:	1.59	1.66	1.09	1.08	1.09	0.97	0.97	0.97	0.03					
ISLll1:	1.55	1.64	1.09	1.07	1.08	0.96	0.96	0.97	0.02	0.02				
IS982B:	1.57	1.64	1.10	1.07	1.08	0.95	0.95	0.96	0.03	0.01	0.01			
ISLla2:	1.59	1.74	1.10	1.09	1.10	0.98	0.98	1.01	0.09	0.08	0.07	0.07		
ISLgar2:	1.57	1.70	1.11	1.12	1.11	0.99	0.99	0.98	0.16	0.16	0.15	0.15	0.16	
ISLgar3:	1.78	1.72	1.25	1.18	1.22	1.05	1.05	1.08	0.91	0.92	0.91	0.90	0.93	0.90
ISLhe1:	1.64	1.64	1.09	1.16	1.09	1.07	1.07	1.12	1.00	0.98	0.97	0.97	1.00	1.02
ISLhe7:	1.54	1.61	1.10	1.16	1.12	1.09	1.09	1.11	1.04	1.02	1.01	1.01	1.05	1.05
ISLhe5:	1.59	1.63	1.12	1.15	1.13	1.09	1.09	1.13	1.02	1.01	1.00	1.00	1.03	1.03
ISLh1:	1.53	1.61	1.11	1.07	1.10	1.01	1.01	1.03	0.93	0.92	0.90	0.90	0.92	0.90
ISLpl4:	1.58	1.53	1.12	1.14	1.16	1.25	1.25	1.21	1.08	1.07	1.06	1.06	1.07	1.05
ISSmu5:	1.65	1.65	1.09	1.10	1.08	1.16	1.16	1.13	1.16	1.13	1.12	1.11	1.12	1.17
ISSa4:	1.60	1.60	1.12	1.17	1.09	1.17	1.17	1.19	1.16	1.14	1.14	1.13	1.15	1.13
ISScr1:	1.62	1.58	1.15	1.22	1.15	1.15	1.15	1.16	1.09	1.07	1.07	1.06	1.08	1.07
ISSpy2:	1.62	1.58	1.12	1.15	1.14	1.12	1.12	1.16	1.09	1.07	1.07	1.06	1.08	1.06
ISSsu12:	1.64	1.64	1.11	1.16	1.10	1.12	1.12	1.16	1.12	1.09	1.09	1.09	1.11	1.13
ISClce1:	1.59	1.64	1.27	1.25	1.27	1.36	1.36	1.27	1.25	1.24	1.24	1.23	1.24	1.20
ISCth1:	1.53	1.53	1.33	1.33	1.34	1.39	1.39	1.39	1.32	1.33	1.30	1.30	1.27	1.29
ISRmsp1:	1.62	1.57	1.34	1.33	1.28	1.40	1.40	1.44	1.28	1.27	1.26	1.25	1.30	1.27
ISNsp1:	1.64	1.77	1.44	1.44	1.41	1.55	1.55	1.70	1.55	1.54	1.54	1.52	1.54	1.54
ISPsma1:	1.60	1.71	1.49	1.49	1.45	1.73	1.73	1.45	1.52	1.48	1.48	1.47	1.55	1.58
ISAba825:	1.61	1.70	1.59	1.53	1.53	1.61	1.61	1.48	1.54	1.51	1.51	1.49	1.57	1.54
ISAba47:	1.57	1.64	1.53	1.52	1.46	1.59	1.59	1.47	1.45	1.43	1.43	1.41	1.48	1.46
ISAba9:	1.61	1.71	1.53	1.55	1.44	1.61	1.61	1.48	1.51	1.50	1.50	1.48	1.56	1.55
ISRa1:	1.66	1.70	1.21	1.25	1.27	1.48	1.48	1.45	1.39	1.37	1.37	1.37	1.37	1.42
ISFba2:	1.70	1.64	1.27	1.30	1.32	1.53	1.53	1.58	1.45	1.42	1.43	1.43	1.42	1.50
IS1187:	1.73	1.87	1.44	1.46	1.47	1.66	1.66	1.62	1.51	1.48	1.50	1.50	1.48	1.50
ISPrsp2:	1.67	1.74	1.35	1.40	1.40	1.55	1.55	1.62	1.49	1.46	1.48	1.48	1.46	1.48
ISPfu3:	1.96	2.01	1.61	1.67	1.58	1.79	1.79	1.60	1.69	1.69	1.69	1.69	1.73	1.69
ISTli1:	1.92	2.03	1.62	1.68	1.59	1.78	1.78	1.66	1.71	1.69	1.69	1.69	1.73	1.69

	ISLgar3:	ISLhe1:	ISLhe7:	ISLhe5:	ISLh1:	ISLpl4:	ISSmu5:	ISSa4:	ISScr1:	ISSpy2:	ISSsu12:
ISLhe1:	1.02										
ISLhe7:	1.12	0.04									
ISLhe5:	1.10	0.12	0.10								
ISLh1:	1.07	0.33	0.28	0.27							
ISLpl4:	1.11	0.75	0.75	0.73	0.71						
ISSmu5:	1.18	0.83	0.81	0.81	0.74	0.72					
ISSa4:	1.13	0.72	0.74	0.72	0.67	0.71	0.52				
ISScr1:	1.14	0.68	0.70	0.69	0.64	0.70	0.50	0.12			
ISSpy2:	1.10	0.69	0.72	0.71	0.64	0.68	0.52	0.19	0.18		
ISSsu12:	1.14	0.77	0.74	0.70	0.67	0.72	0.63	0.29	0.25	0.24	
ISClce1:	1.36	1.20	1.18	1.17	1.09	1.23	1.25	1.24	1.26	1.21	1.19
ISCth1:	1.39	1.30	1.26	1.27	1.17	1.37	1.29	1.33	1.31	1.26	1.33
ISRmsp1:	1.33	1.31	1.22	1.22	1.13	1.37	1.27	1.29	1.27	1.22	1.24
ISNsp1:	1.39	1.35	1.34	1.37	1.34	1.41	1.39	1.35	1.32	1.34	1.32
ISPsma1:	1.55	1.43	1.38	1.38	1.41	1.51	1.32	1.42	1.31	1.37	1.37
ISAba825:	1.57	1.41	1.42	1.45	1.36	1.57	1.42	1.46	1.40	1.41	1.49
ISAba47:	1.48	1.41	1.42	1.40	1.33	1.57	1.38	1.40	1.34	1.35	1.35
ISAba9:	1.56	1.37	1.40	1.42	1.34	1.57	1.36	1.40	1.32	1.31	1.35
ISRa1:	1.48	1.30	1.30	1.30	1.28	1.40	1.32	1.24	1.27	1.27	1.24
ISFba2:	1.64	1.31	1.33	1.31	1.24	1.43	1.39	1.26	1.35	1.29	1.21
IS1187:	1.58	1.36	1.40	1.36	1.38	1.57	1.58	1.40	1.43	1.44	1.37
ISPrsp2:	1.59	1.29	1.33	1.32	1.31	1.41	1.39	1.28	1.34	1.30	1.32
ISPfu3:	1.69	1.68	1.54	1.52	1.61	1.56	1.52	1.51	1.51	1.50	1.45
ISTli1:	1.65	1.69	1.56	1.55	1.68	1.59	1.55	1.51	1.53	1.54	1.51

	ISClce1:	ISCth1:	ISRmsp1:	ISNsp1:	ISPsma1:	ISAba825:	ISAba47:	ISAba9:	ISRa1:	ISFba2:	IS1187:	ISPrsp2:	ISPfu3:	ISTli1:
ISCth1:	0.49													
ISRmsp1:	0.72	0.68												
ISNsp1:	1.21	1.28	1.28											
ISPsma1:	1.11	1.25	1.27	0.78										
ISAba825:	1.20	1.22	1.31	0.76	0.48									
ISAba47:	1.14	1.17	1.28	0.76	0.48	0.27								
ISAba9:	1.22	1.25	1.28	0.79	0.48	0.28	0.14							
ISRa1:	1.05	1.14	1.19	1.42	1.39	1.42	1.39	1.45						
ISFba2:	1.13	1.28	1.21	1.37	1.53	1.45	1.37	1.44	0.59					
IS1187:	1.18	1.28	1.29	1.43	1.42	1.39	1.33	1.42	0.77	0.69				
ISPrsp2:	1.21	1.32	1.25	1.41	1.38	1.43	1.40	1.42	0.73	0.71	0.37			
ISPfu3:	1.47	1.47	1.58	1.78	1.66	1.74	1.75	1.74	1.52	1.44	1.54	1.62		
ISTli1:	1.53	1.53	1.57	1.79	1.60	1.76	1.78	1.74	1.58	1.51	1.59	1.66	0.09	

Suppelemtary Table S1
